# Implications of vitamin D levels or status for mortality in rheumatoid arthritis: analysis of 2001-2018 data from the National Health and Nutrition Examination Survey

**DOI:** 10.3389/fimmu.2024.1425119

**Published:** 2024-10-09

**Authors:** Yalin Feng, Ping Zhu, Dandan Yan, Xu Wang, Caiyun Chen, Zhongyuan Zhang, Yian Tian, Jiajia Wang, Shanshan Liu, Ju Li, Deqian Meng, Kai Wang

**Affiliations:** ^1^ Department of Laboratory, The Affiliated Huaian No.1 People’s Hospital of Nanjing Medical University, Huaian, China; ^2^ Department of Endocrinology, The Affiliated Chuzhou Hospital of Traditional Chinese Medicine of Jiangsu College of Nursing, Huaian, China; ^3^ Department of Rheumatology and Immunology, The Affiliated Huaian No.1 People’s Hospital of Nanjing Medical University, Huaian, China; ^4^ Huaian Key Laboratory of Autoimmune Diseases, Huaian, China

**Keywords:** vitamin D, vitamin D levels, vitamin D status, mortality, rheumatoid arthritis

## Abstract

**Background:**

Inadequate levels of vitamin D (VitD) have been linked to increased rates of various health conditions and mortality. However, little is known about the relationship between mortality outcomes and 25-hydroxyvitamin D [25(OH)D] levels in individuals with rheumatoid arthritis (RA). This study aimed to examine this association using data from the National Health and Nutrition Examination Survey.

**Methods:**

A cohort of 2,290 individuals aged 20 to 85 years with RA was analyzed. Lower 25(OH)D levels were inversely associated with all-cause mortality, with a hazard ratio (HR) of 0.91 (0.87 to 0.96) per 10 nmol/L increase. Comparatively, the HR for the VitD insufficiency group was 0.64 (0.50 to 0.83), and for the VitD sufficiency group, it was 0.60 (0.44 to 0.80), both compared to the VitD deficiency group. Cause-specific analysis showed that higher 25(OH)D levels were associated with reduced mortality from heart disease (HR: 0.88, 0.82 to 0.95) and malignant neoplasms (HR: 0.86, 0.79 to 0.94). No significant correlation was found between 25(OH)D levels and cause-specific mortalities for other conditions.

**Results:**

Stratified by gender, the HR for males was 0.92 (0.85 to 0.99) and for females was 0.91 (0.86 to 0.98) per 10 nmol/L increase in 25(OH)D levels. Among individuals aged 20-59 years, no significant correlation was observed, while for those aged 60 years and older, the HR was 0.86 (0.82 to 0.90) per 10 nmol/L increase. Nonlinear analysis identified a sharp increase in HR below 59.95 nmol/L, while HR remained below 1 for 25(OH)D levels above 59.95 nmol/L.

**Conclusion:**

This study reveals a strong negative correlation between 25(OH)D levels and overall mortality in individuals with RA. Notably, this association is particularly significant for mortality related to heart disease and malignant neoplasms. Targeted VitD supplementation should be emphasized, especially in individuals aged 60 years and older with RA. The proposed minimum threshold for adequate 25(OH)D levels in the RA population is 60 nmol/L.

## Introduction

Rheumatoid arthritis (RA) is a chronic autoimmune disorder characterized by persistent inflammation primarily targeting the joints, resulting in discomfort, swelling, and impaired physical functioning. The global prevalence of RA is approximately 0.5%, with a higher susceptibility observed in women (1). Despite extensive research, the precise etiology of RA remains incompletely understood, though evidence suggests that immune system dysregulation and genetic predisposition contribute significantly to its development (2). Environmental factors such as UV exposure, air pollution, and dietary habits, as well as lifestyle choices including smoking, alcohol consumption, and physical activity levels have been implicated in the development and progression of RA (3, 4).

The association between Vitamin D (VitD) deficiency and autoimmune diseases, including RA, has garnered increased attention in recent years (5). Research conducted in various countries has consistently demonstrated a notable prevalence of VitD deficiency among individuals diagnosed with RA (6–9). Furthermore, lower levels of VitD have been found to correlate with more severe clinical manifestations of RA (10).

VitD deficiency is prevalent among RA patients across different ethnic groups and geographical regions. In the United States, a study found that 84% of African American RA patients had suboptimal VitD levels (11). Similar trends have been observed in other populations, with another study reporting that 84% of Caucasian RA patients had suboptimal VitD levels (12). The global nature of this issue is further emphasized by studies from various countries. In Saudi Arabia, a cross-sectional study revealed that among RA patients, 42.7% had insufficient VitD levels, 22.3% had a deficiency, and 15.5% had severe deficiency (7). This study also found significant correlations between VitD levels and disease activity measures. Research from Iran reported that 34.8% of RA patients had insufficient VitD levels. Importantly, this study demonstrated a significant relationship between serum VitD levels and disease severity (9). A meta-analysis of studies from India showed that 76.1% of RA patients were VitD deficient (13). These findings collectively underscore that VitD deficiency in RA patients is a global phenomenon, not limited to any particular ethnic group or geographical region.

Despite ongoing research, the precise nature of the relationship between VitD and RA remains incompletely understood, leaving uncertainty regarding whether VitD is a causative factor or an outcome of the condition (14). A recent meta-analysis failed to establish definitive evidence linking 25-hydroxyvitamin D [25(OH)D] levels with susceptibility to RA (15). However, a study conducted in China involving 493 RA patients revealed a genetic association between VitD metabolism pathway genes (CYP2R1 and CYP27B1) and the genetic background of RA. Moreover, a correlation was observed between alterations in methylation levels of VitD receptor (VDR) and CYP27B1, as well as an increased susceptibility to RA, indicating the potential involvement of dysregulated VitD metabolism in the onset of RA (16).

Over the past few years, researchers have increasingly focused on investigating the relationship between VitD and both all-cause mortality and cause-specific mortality. Serum levels of 25(OH)D have consistently demonstrated a negative correlation with all-cause mortality, as well as mortality specifically attributed to cardiovascular diseases (CVD), cancer, and respiratory system diseases (17–20). It is worth noting that individuals with RA often exhibit lower 25(OH)D levels and higher mortality rates compared to the general population (21, 22). A study conducted in Mexico revealed a significant association between insufficient 25(OH)D levels and an elevated risk of suicide in individuals with RA (23). Nonetheless, there is currently a lack of published studies investigating the relationship between mortality and 25(OH)D levels within the RA population.

The therapeutic potential of VitD in RA has been evidenced by previous studies (14, 24). In addition to enhancing skeletal health in RA patients, VitD has shown the ability to reduce DAS28 by modulating the proportion of regulatory T cells (Tregs) in this population (25, 26). However, further investigations are warranted to determine the optimal dosage, treatment duration, and identify the specific patient subgroups that would derive the greatest benefits from such interventions.

The primary objective of this study, utilizing a comprehensive database, is to examine the association between mortality and 25(OH)D levels within the RA population. By analyzing extensive clinical data and conducting long-term follow-up, we aim to obtain a comprehensive understanding of the impact of VitD in the RA population and provide scientific evidence for prevention and treatment strategies. Additionally, exploring the epidemiology and burden of RA will contribute to a better comprehension of the disease’s impact on patients and serve as a reference for public health interventions.

## Methods

### Study population

The data used in this study originates from the National Health and Nutrition Examination Survey (NHANES), which has been conducting comprehensive and representative surveys nationwide every two years since 1999. NHANES employs complex, stratified, multi-stage probability sampling techniques to ensure accuracy. The surveys encompass household interviews and physical examinations conducted at mobile examination centers. In this study, data from nine NHANES survey cycles spanning 2001 to 2018 were employed. The dataset encompasses various factors, such as sociodemographic characteristics, body mass index, comorbidities, and VitD information, which were amalgamated into a unified dataset. Participants without mortality data were excluded from the analysis (**Supplementary Figure 1**).

### Statistical analyses

Descriptive statistics were used to summarize the sociodemographic characteristics of the study population. VitD status were defined based on 25(OH)D levels as deficiency (< 50 nmol/L), insufficiency (50 - 75 nmol/L), or sufficiency (> 75 nmol/L) (27, 28). Median values and interquartile ranges (IQR) were utilized to summarize continuous variables, while frequencies and percentages were used to present categorical variables. Statistical tests appropriate for each variable type were utilized to assess differences in various characteristics. Specifically, chi-square tests were employed for categorical variables, and Kruskal-Wallis tests were used for continuous variables.

Cox proportional hazards regression models were employed to investigate the association between 25(OH)D levels and both all-cause mortality and cause-specific mortality. VitD status was included as a categorical variable in the model. Additionally, to investigate the association between all-cause mortality and 25(OH)D levels, subgroup analyses were performed, focusing on specific subgroups for a more targeted examination, such as age and gender. In these analyses, 25(OH)D levels was included as a continuous variable (per 10 nmol/L). To compare mortality rates among various subgroups, hazard ratios (HR) and corresponding 95% confidence intervals (CI) were computed.

To explore possible nonlinear relationships between 25(OH)D levels and mortality, restricted cubic splines (RCS) with Cox regression models were utilized. We have tested knots between 3 and 7 and selected the model with the lowest Akaike information criterion value for the RCS analysis. Adjustments were made for a range of covariates, including sex, age, race, annual household income, marital status, education level, body mass index (BMI), diabetes, hypertension, weak/failing kidneys, and total cholesterol. The statistical analyses were conducted utilizing R version 4.1.0.

## Results

### Study participants

After excluding 7,102 participants without VitD data, 24,207 participants without mortality data, and 45,188 participants who were not diagnosed with RA, a total of 2,290 participants were included in the study. **Table 1** presents the weighted baseline characteristics of the participants categorized by status (unweighted results are presented in **Supplementary Table 1**). Compared to deceased individuals with RA, individuals with RA who were still alive had higher levels of 25(OH)D. The baseline characteristics of individuals with RA, categorized by their VitD status, are presented in **Supplementary Tables 2** and **3**, showcasing both unweighted and weighted results. Non-Hispanic White individuals had better VitD status compared to other racial groups. The married/cohabiting group had better VitD status compared to the other two groups. Annual household income and education levels were positively correlated with 25(OH)D levels (**Supplementary Figure 2**). BMI was negatively correlated with 25(OH)D levels (**Supplementary Figure 2**).

**Table 1:** Demographic characteristics of individuals with RA according to status, weighted.

Characteristic	Assumed alive	Assumed deceased	p-value
	(N=6362191)	(N=1818297)	
Sex = female (%)	3792480 (59.6)	1011732 (55.6)	0.125
Age	55.00 (45.00, 64.00)	71.00 (60.00, 79.00)	**<0.001**
Race (%)			<0.001
Mexican American	478848 (7.5)	69616 (3.8)	
Other Hispanic	363797 (5.7)	49917 (2.7)	
Non-Hispanic White	4043359 (63.6)	1391776 (76.5)	
Non-Hispanic Black	1077391 (16.9)	257166 (14.1)	
Other Race	398794 (6.3)	49819 (2.7)	
25(OH)D (nmol/L)	66.50 (50.13, 84.55)	60.60 (41.51, 77.21)	**<0.001**
Months of follow-up	101.06 (51.00, 157.00)	72.00 (34.00, 119.00)	**<0.001**
Annual household income (%)			<0.001
Under $44,999	3411020 (53.6)	1373444 (75.5)	
$45,000 to $74,999	1769551 (27.8)	383318 (21.1)	
$75,000 and over	1181620 (18.6)	61534 (3.4)	
Marital status (%)			<0.001
Married/cohabiting	4064740 (63.9)	847536 (46.6)	
Widowed/divorced/separated	1781102 (28.0)	881905 (48.5)	
Never married	516348 (8.1)	88855 (4.9)	
Education level (%)			<0.001
Under high school	1368188 (21.5)	668718 (36.8)	
High school or equivalent	1718629 (27.0)	530548 (29.2)	
Above high school	3275373 (51.5)	619030 (34.0)	
BMI	29.80 (25.27, 34.80)	28.48 (24.51, 32.64)	**<0.001**
Diabetes (%)			0.001
No	5078261 (79.8)	1263883 (69.5)	
Borderline	180937 (2.8)	71952 (4.0)	
Yes	1102993 (17.3)	482462 (26.5)	
Hypertension = Yes (%)	3120571 (49.0)	1281196 (70.5)	**<0.001**
Weak/failing kidneys = Yes (%)	414799 (6.5)	142652 (7.8)	0.373
Total Cholesterol (mmol/L)	5.04 (4.40, 5.74)	4.92 (4.19, 5.82)	0.423

25(OH)D, 25-hydroxyvitamin D; BMI, Body mass index. The bold values means statistical significance.

### Cox proportional hazards regression

**Figure 1:**
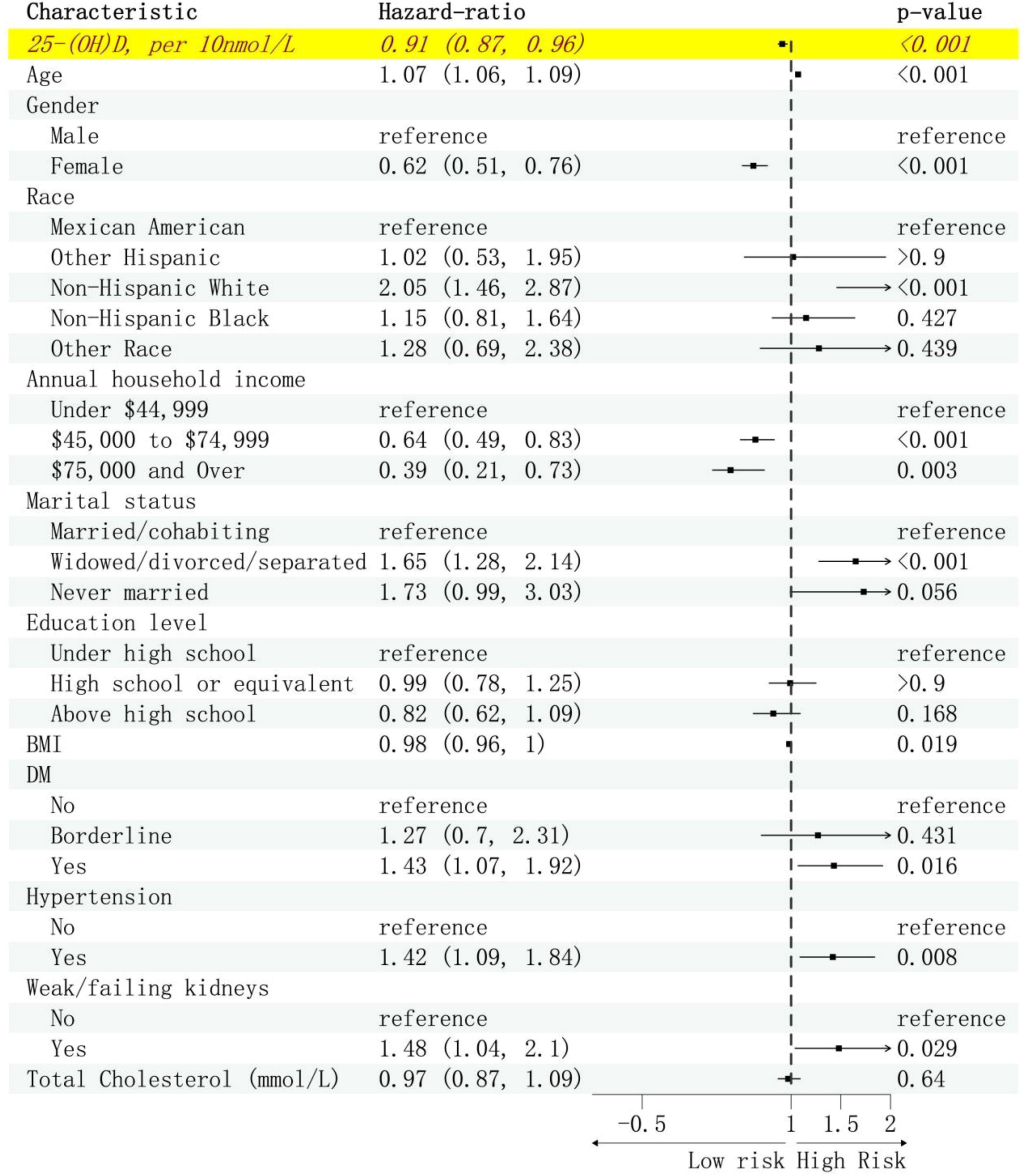
Hazard ratios (95% CI) of 25(OH)D and all covariates, weighted. The model was adjusted for age, sex, race, annual household income, marital status, education level, BMI, diabetes, hypertension, weak/failing kidneys, and total cholesterol.

Over a follow-up period of 227 months (with a median of 91 months), a total of 586 deaths (weighted: 1,818,297) were identified. **Figure 1** displays the association between 25(OH)D levels, covariates, and all-cause mortality. A robust negative correlation was observed between VitD and all-cause mortality, with a predicted HR of 0.91 (weighted: 0.87 to 0.96) per 10 nmol/L increase in 25(OH)D levels. **Supplementary Figure 3** presents the unweighted results. Regarding cause-specific mortalities, the HR per 10 nmol/L increase in 25(OH)D levels was 0.88 (weighted: 0.82 to 0.95) for diseases of heart, 0.86 (weighted: 0.79 to 0.94) for malignant neoplasms and 0.92 (weighted: 0.85 to 0.99) for all other causes. However, no significant correlation was found between 25(OH)D levels and cause-specific mortalities from chronic lower respiratory diseases, influenza and pneumonia, accidents, cerebrovascular diseases, diabetes, Alzheimer’s disease, nephritis, nephrotic syndrome and nephrosis diseases (**Table 2**). **Supplementary Table 4** provides the association between VitD status and all-cause mortality. Compared to the VitD deficiency group, the HR for the VitD insufficiency group was 0.64 (weighted: 0.50 to 0.83), and for the VitD sufficiency group, it was 0.60 (weighted: 0.44 to 0.80).

**Table 2:** According to 25(OH)D levels, the HR (95% CI) of all-cause mortality among individuals with RA.

Characteristic	Unweighted		Weighted	
Cause specific mortality	HR per 10nmol/L (95% CI)	p-value	HR per 10nmol/L (95% CI)	p-value
Diseases of heart	0.88 (0.82, 0.95)	**<0.001**	0.87 (0.79, 0.97)	**0.012**
Malignant neoplasms	0.86 (0.79, 0.94)	**<0.001**	0.86 (0.76, 0.97)	**0.011**
Chronic lower respiratory diseases	1.10 (0.97, 1.24)	0.142	1.05 (0.89, 1.24)	0.546
Accidents	1.04 (0.70, 1.53)	0.846	1.08 (0.66, 1.76)	0.766
Cerebrovascular diseases	0.95 (0.81, 1.12)	0.568	0.94 (0.79, 1.12)	0.486
Alzheimer’s disease	1.11 (0.91, 1.36)	0.308	1.15 (0.89, 1.50)	0.286
Diabetes mellitus	0.93 (0.74, 1.17)	0.545	0.95 (0.67, 1.35)	0.772
Influenza and pneumonia	1.07 (0.83, 1.38)	0.579	1.08 (0.88, 1.33)	0.446
Nephritis, nephrotic syndrome and nephrosis	0.84 (0.63, 1.11)	0.224	0.88 (0.70, 1.11)	0.273
All other causes	0.94 (0.87, 1.00)	0.051	0.92 (0.85, 0.99)	**0.026**

HR, Hazard ratio; CI, Confidence interval; 25(OH)D, 25-hydroxyvitamin D. The bold values means statistical significance. HRs were adjusted for age, sex, race, annual household income, marital status, education level, BMI, diabetes, hypertension, weak/failing kidneys, and total cholesterol.

Furthermore, we conducted subgroup analyses of the association between 25(OH)D levels and all-cause mortality based on selected features, including age and sex (**Table 3**). The HR for per 10 nmol/L increase in 25(OH)D levels was 0.92 (weighted, 0.85 to 0.99) for males and 0.91 (weighted, 0.86 to 0.98) for females. There was no significant correlation between 25(OH)D levels and mortality among individuals aged 20-59 years. However, among individuals aged 60 years and above, the HR for per 10 nmol/L increase in 25(OH)D levels was 0.86 (weighted, 0.82 to 0.90).

**Table 3:** According to 25(OH)D levels, the HR (95% CI) of all-cause mortality in different sex and age intervals.

Characteristic	Unweighted		Weighted	
	HR per 10nmol/L (95% CI)	p-value	HR per 10nmol/L (95% CI)	p-value
Sex				
Male	0.93 (0.88, 0.98)	**0.009**	0.92 (0.85, 0.99)	**0.034**
Female	0.91 (0.86, 0.96)	**<0.001**	0.91 (0.86, 0.98)	**<0.001**
Age				
20-39	1.58 (0.68, 3.66)	0.285	3.50 (0.12, 99.30)	0.464
40-59	0.92 (0.83, 1.03)	0.15	0.93 (0.81, 1.07)	0.324
60 and over	0.92 (0.88, 0.96)	**<0.001**	0.92 (0.86, 0.97)	**<0.001**

HR, Hazard ratio; CI, Confidence interval; 25(OH)D, 25-hydroxyvitamin D. The bold values means statistical significance. HRs were adjusted for age, sex, race, annual household income, marital status, education level, BMI, diabetes, hypertension, weak/failing kidneys, and total cholesterol.

### RCS with cox regression

**Figure 2:**
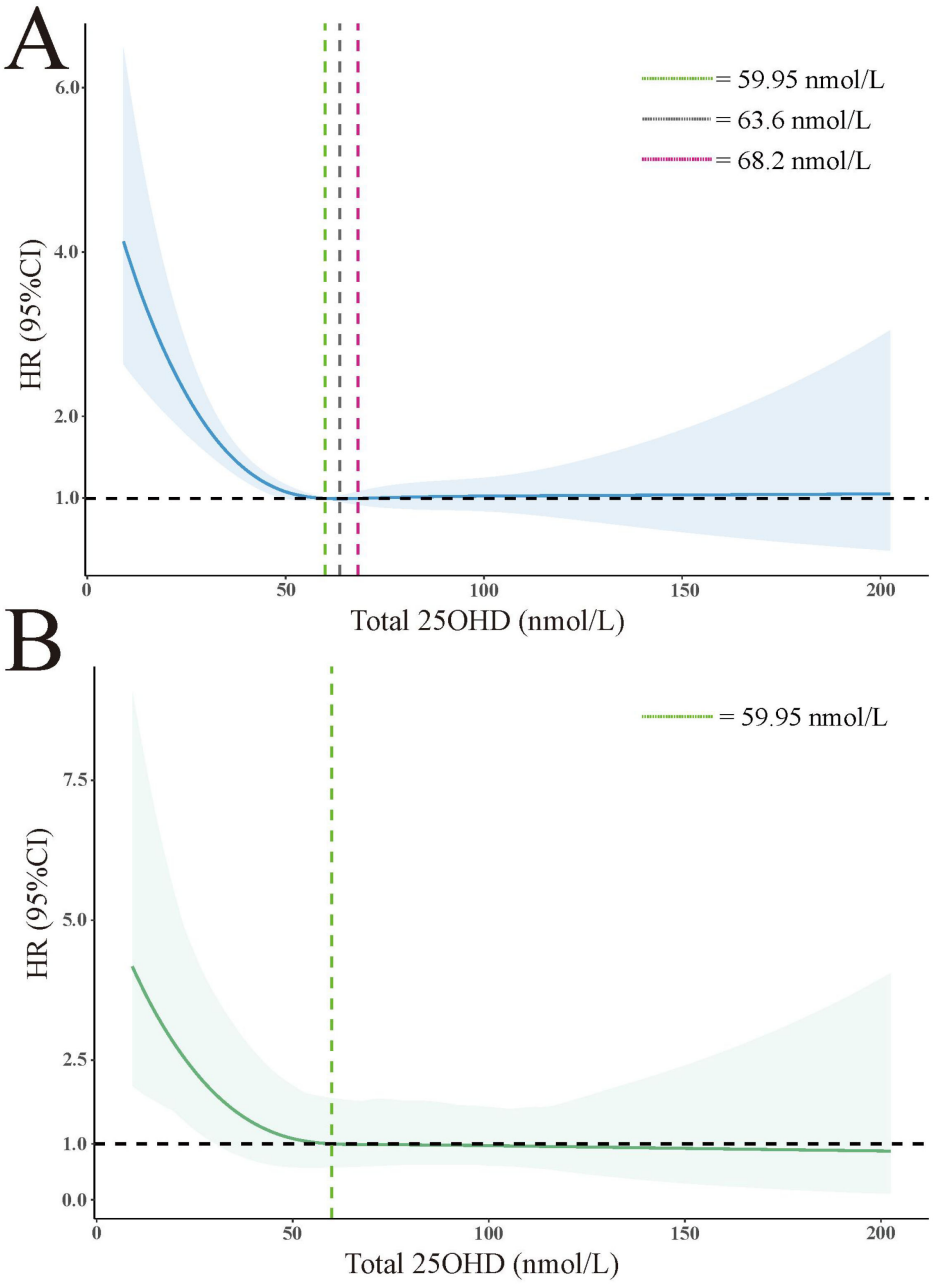
Association of total 25(OH)D level with all-cause mortality in all participants. **(A)** unweighted. **(B)** weighted. The HRs are represented by solid lines, while the 95% confidence intervals are depicted by shaded areas. Knots are positioned at the 5th, 27.5th, 50th, 72.5th, and 95th centiles of the 25(OH)D distribution. All models were adjusted for age, sex, race, annual household income, marital status, education level, BMI, diabetes, hypertension, weak/failing kidneys, and total cholesterol.

To model the association between 25(OH)D levels and all-cause mortality, we applied RCS to capture the relationship in a flexible and visual manner. Specifically, we used RCS with 4 knots positioned at the 5th, 35th, 65th, and 95th centiles. **Figure 2A** illustrates the J-shaped pattern observed in the relationship between 25(OH)D levels and all-cause mortality. Notably, within the 25(OH)D levels range of 59.95 to 68.2 nmol/L, there was a significant decrease in risk, with the lowest risk observed at approximately 63.6 nmol/L (unweighted). Weighted results are presented in **Figure 2B**, illustrating a notable increase in mortality risk for 25(OH)D levels below 59.95 nmol/L, while mortality rates consistently remained below 1.0 for levels above 59.95 nmol/L, indicating a decrease in risk.

## Discussion

The observed association between serum 25(OH)D levels and mortality in individuals with RA aligns with prior research. We identified a negative correlation between serum 25(OH)D levels and all-cause mortality, with the VitD sufficiency group (> 75 nmol/L) showing the most pronounced negative correlation. These findings imply that individuals with RA may require higher levels of 25(OH)D to optimize their health status. Previous investigations have also established a negative correlation between 25(OH)D levels and mortality rates associated with CVD, cancer, and respiratory system diseases (17–20). Notably, a study conducted in Germany involving 5899 participants aged 50-75 years identified the most robust association with mortality related to respiratory system diseases (20). Moreover, a recent meta-analysis has indicated that decreased serum 25(OH)D levels are not only linked to elevated overall cardiovascular events and cardiovascular mortality but also to an increased risk of heart failure (29). Another meta-analysis demonstrated that although VitD supplementation did not reduce the overall incidence of cancer, it significantly lowered overall cancer mortality (30). Additionally, a prospective cohort study involving 493 patients in the United States showed that pancreatic cancer patients with sufficient 25(OH)D levels before diagnosis experienced an extended overall survival period (31).

Our findings both align with and differ from a recent study by Cai et al. (2023) on VitD and mortality in RA patients (32). While both studies found an inverse association between 25(OH)D levels and all-cause mortality, we identified a higher threshold for mortality risk reduction. This discrepancy may be due to our larger sample size, extended study period, and different variable selection in our models. Our additional analyses of cause-specific mortality and age stratification provide further insights, particularly for patients aged 60 and above. These differences underscore the need for further research to establish optimal VitD levels for RA patients.

The findings of our study emphasize the distinctions observed between individuals with RA and other populations. Specifically, among individuals with RA, a robust inverse correlation is evident between serum 25(OH)D levels and all-cause mortality, particularly mortality attributed to cardiovascular diseases and malignant neoplasms. However, no significant association is observed between 25(OH)D levels and specific causes of death such as chronic lower respiratory diseases, accidents, cerebrovascular diseases, diabetes, Alzheimer’s disease, nephritis, nephrotic syndrome, and nephrosis. Furthermore, there is no significant correlation between 25(OH)D levels and all-cause mortality among individuals aged 20-59 years. Nevertheless, for individuals with RA aged 60 years and above, higher levels of 25(OH)D exhibit a link to reduced all-cause mortality, underscoring the significance of VitD supplementation in this specific population. While our study did not find a significant correlation between 25(OH)D levels and all-cause mortality among individuals aged 20 to 59 years, this does not negate the importance of maintaining adequate VitD levels in this age group. VitD plays crucial roles in bone health, immune function, and other physiological processes throughout life (27). However, the long-term effects of VitD supplementation in younger adults on mortality outcomes in later life remain unclear. To our knowledge, no longitudinal studies have directly addressed whether VitD supplementation in people aged 20 to 59 can prevent all-cause mortality when they reach 60 and above. This represents an important area for future research. Nonlinear analysis reveals an L-shaped relationship between 25(OH)D levels and all-cause mortality. The risk of mortality significantly increases for 25(OH)D levels below 59.95 nmol/L, while the HR for levels above 59.95 nmol/L consistently remains below 1. In summary, our findings highlight individuals aged 60 and above with RA as a crucial target group for VitD supplementation, with a minimum threshold of 59.95 nmol/L for 25(OH)D levels among individuals with RA.

There are multiple potential mechanisms through which the mortality rate among patients with RA can be influenced by levels of 25(OH)D. It is noteworthy that a negative correlation exists between levels of 25(OH)D and the severity of the disease in RA patients, indicating a direct contribution of lower 25(OH)D levels to mortality associated with RA (6–9). Additionally, there is an inverse association between levels of 25(OH)D and severe muscle wasting, reduced physical fitness, and decreased skeletal muscle mass in RA patients, thereby increasing the risk of osteoporotic fractures and mortality (10, 33). Moreover, insufficient levels of 25(OH)D are associated with an elevated risk of suicide in individuals with RA (23).

The pathogenesis of RA involves the interplay between T cells, B cells, and their interactions with pro-inflammatory cytokines (34). T helper (Th)-1 and Th17 cells induce pro-inflammatory cytokines such as interferon–γ, tumor necrosis factor-α, interleukin (IL)-2, IL-17, and IL-22, leading to the destruction of cartilage and erosion of bones (35, 36). Conversely, Th2 cells and cytokines have a suppressive effect on the immune progression of RA. Th2 cells mainly secrete IL-4, IL-5, IL-10, and IL-13, with IL-4 and IL-13 capable of alleviating synovial inflammation, inhibiting osteoclastogenesis, and improving bone erosion (37–39). In addition to the Th1/Th2/Th17 imbalance, RA is characterized by reduced Treg activity, including a decrease in Treg cell numbers and an imbalance between Treg and Th17 cells (40–42). The imbalance between Treg and Th17 cells aggravates arthritis and bone destruction by promoting the expression of RANKL on synovial fibroblasts (43).

Apart from its impact on the musculoskeletal system, the immunomodulatory properties of VitD are its most notable characteristic. The active form of VitD [1,25(OH)2D] effectively regulates the phenotype and physiology of various cell types. It enhances the function of Th2 and Treg cells while inhibiting the function of Th1 and Th17 cells. *In vitro*, it demonstrates significant anti-inflammatory effects on CD4^+^ and CD8^+^ T cells and *in vivo*, it suppresses the production of cytokines associated with Th1 and Th17 cells (44). The autocrine VitD signaling can trigger the contraction of the Th1 cell response, thereby shutting down the pro-inflammatory program of Th1 cells (45). Consequently, the pathogenesis of RA involves complex immune regulation, and VitD deficiency contributes to the pro-inflammatory state in RA through its impact on Th cell subsets (Th1, Th2, Treg, and Th17 cells) and their secreted cytokines.

In the context of cancer, 1,25(OH)2D exhibits inhibitory effects on the proliferation of various normal and tumor cells while promoting their terminal differentiation. This action primarily occurs through the antagonism of signaling pathways, including Wnt/β-catenin, epidermal growth factor, and transforming growth factor-β, which suppress epithelial-mesenchymal transition (46–49). Additionally, VitD can interrupt the cell cycle by directly altering cell cycle regulators that induce cell cycle arrest, thereby reducing tumor cell proliferation (50). The anticancer effects of VitD, besides inhibiting tumor cell proliferation, may also arise from controlling the growth and differentiation of the immune system (51).

VitD insufficiency is linked to various CVD, including vascular dysfunction, atherosclerosis, left ventricular hypertrophy, hypertension, and dyslipidemia (52–55). The potential mechanisms through which VitD deficiency elevates the risk of CVD are associated with the activation of VDR. VitD exerts diverse cardiovascular effects by activating VDR in cardiomyocytes and endothelial cells. It also influences the renin-angiotensin-aldosterone system, energy expenditure, adiposity, and pancreatic cell function (56). Studies in VDR knockout mice have shown that these mice display hypertension signs, including enhanced activity of the renin-angiotensin-aldosterone system, as well as cardiac hypertrophy characterized by an increased ratio of heart weight to body weight and elevated expression of natriuretic peptides (57, 58).

Our study represents the primary inquiry into the association between levels of 25(OH)D and both all-cause mortality and cause-specific mortality among individuals affected by RA. In this investigation, we have utilized the NHANES database, which incorporates a sophisticated sampling methodology and includes participants spanning a wide age range and diverse ethnic backgrounds. This comprehensive approach ensures a diverse participant pool and augments the external validity of our findings. To mitigate the impact of confounding variables, we have considered various covariates that may potentially influence serum 25(OH)D levels. By doing so, we have minimized the influence of confounding factors and gained confidence in the reliability of our statistical outcomes.

However, it is important to acknowledge the limitations of our study. A primary limitation is the reliance on self-reported data for RA classification in the NHANES dataset. Participants who reported being told by a health professional that they had RA were classified in the RA group. This method may have led to misclassification, potentially including other forms of arthritis or related conditions. The lack of clinical verification of RA diagnoses could impact the accuracy of our findings. Additionally, the NHANES dataset lacks detailed clinical information specific to RA, such as DAS28 index or number of swollen joints. Another significant limitation is the lack of data on VitD intake, both from dietary sources and supplements. The NHANES dataset, while comprehensive in many aspects, does not provide specific information on dietary VitD consumption or VitD supplementation. This limitation restricts our ability to provide a comprehensive understanding of the factors influencing VitD status in RA patients and may impact the interpretation of our results. The sample size of individuals with RA might be limited, potentially restricting our ability to conduct comprehensive analyses. Despite NHANES’s sophisticated sampling design, selection bias cannot be entirely ruled out. These limitations should be considered when interpreting the study results. Future studies would benefit from more rigorous clinical classification of RA, inclusion of RA-specific clinical data, and detailed data on both dietary and supplemental VitD intake to address these gaps and provide a more complete picture of VitD status in RA.

In summary, we have observed a robust inverse association between levels of 25(OH)D and both all-cause mortality and cause-specific mortality in individuals diagnosed with RA. Specifically, this negative correlation between 25(OH)D levels and all-cause mortality was evident only among individuals aged 60 or older with RA. Notably, a noteworthy increase in HR was identified when 25(OH)D levels fell below 59.95 nmol/L, while mortality rates consistently remained below 1 above this threshold, indicating a decrease in risk. Based on these significant findings, we propose the adoption of routine screening for 25(OH)D levels within the RA-affected population, incorporating age stratification, particularly emphasizing individuals aged 60 or older, to facilitate appropriate VitD supplementation. Furthermore, we suggest considering a minimum threshold of 60 nmol/L for 25(OH)D levels among individuals with RA.

## Data Availability

The data used in this study are publicly available from the NHANES, conducted by the National Center for Health Statistics (NCHS) of the Centers for Disease Control and Prevention (CDC). These data can be accessed through the CDC’s NHANES website: https://wwwn.cdc.gov/nchs/nhanes/Default.aspx.
