# Supplementary material for: Implications of vitamin D levels or status for mortality in rheumatoid arthritis: analysis of 2001-2018 data from the National Health and Nutrition Examination Survey
Source: Front Immunol. 2024 Oct 9;15:1425119. doi: 10.3389/fimmu.2024.1425119 (PMC11496074; doi:10.3389/fimmu.2024.1425119)
Supplement: Supplementary file 1 [file DataSheet1.docx]

**The Determination of Vitamin D**

According to the NHANES data collection guidelines, serum specimens were collected using regular red-top or serum-separator Vacutainers™. During 2001-2006, 25-Hydroxyvitamin D [25(OH)D] levels were measured using the Diasorin RIA method. The Diasorin (formerly Incstar) 25-Hydroxyvitamin D (25OHD) assay consists of a two-step procedure. The first procedure involves an extraction of 25OHD metabolites from serum with acetonitrile. Following extraction, the sample is assayed by using an equilibrium RIA procedure. The RIA method is based on an antibody with specificity to several mono- and di-hydroxyvitamin D metabolites. The sample, antibody, and tracer are incubated for 90 min at 20-25 °C. Phase separation is accomplished after a 20-minute incubation at 20-25 °C with a second antibody-precipitating complex. A buffer is added after this incubation and prior to centrifugation to reduce non-specific binding.

During 2007-2018, Ultra-high-performance liquid chromatography–tandem mass spectrometry (UHPLC-MS/MS) was used to quantitatively detect 25(OH)D in human serum. 25(OH)D assay consists of a two-step procedure. The first procedure involves an extraction of 25(OH)D metabolites from serum with acetonitrile. Following extraction, the sample is assayed by using an equilibrium RIA procedure. The RIA method is based on an antibody with specificity to several mono- and di-hydroxyvitamin D metabolites. The sample, antibody, and tracer are incubated for 90 min at 20-25 °C. Phase separation is accomplished after a 20-minute incubation at 20-25 °C with a second antibody-precipitating complex. A buffer is added after this incubation and prior to centrifugation to reduce non-specific binding.

**Other Covariates**

Questionnaire information included general demographic characteristics and general life behaviors. General demographic characteristics included age, sex, race, education level, marital status, annual household income, diabetes, hypertension, and weak/failing kidneys. The information was based on participants’ self-reports. In addition, body mass index (BMI) was calculated by dividing the weight (kg) by height (meters) squared. Cholesterol was measured using the Beckman UniCel® DxC 800 Synchron and Beckman UniCel® DxC 660i Synchron Access Clinical Systems. The setting of the interview was a private room in the Mobile Examination Center (MEC). Each MEC dietary interview room contained a standard set of measuring guides.

**Measurements of RA**

The assessment of RA was determined by self-report questionnaire questions. These questions included "Has a doctor or other health professional ever told you that you had arthritis?" and "Which type of arthritis was it?". Participants who answered "RA" to the latter question were classified in the RA group, while those who answered "no" to the former question and "osteoarthritis, degenerative arthritis, psoriasis, and others" to the latter question were classified in the non-RA group.


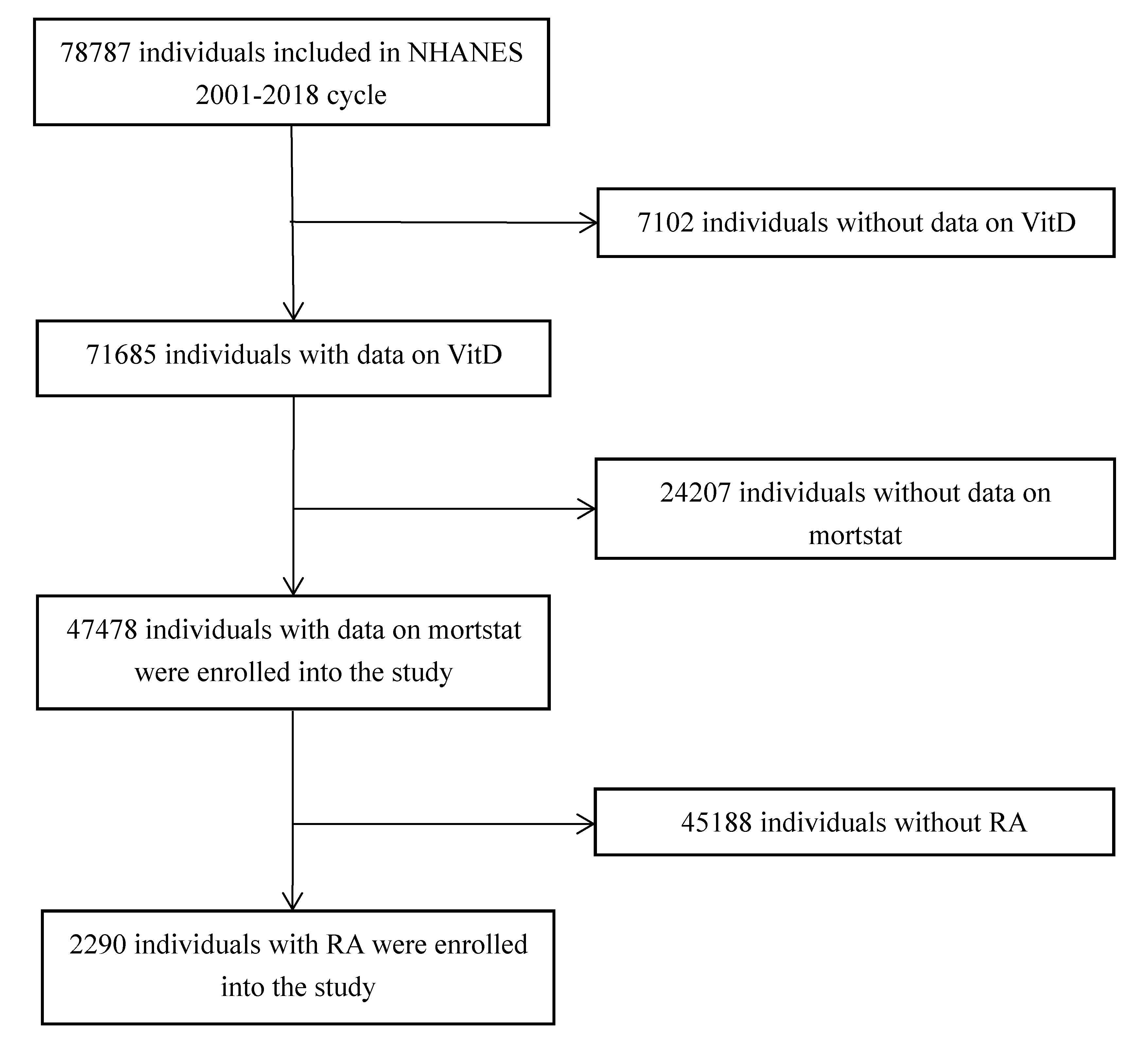


**Supplementary Figure 1.** Flow chart of participants enrolled into the study.


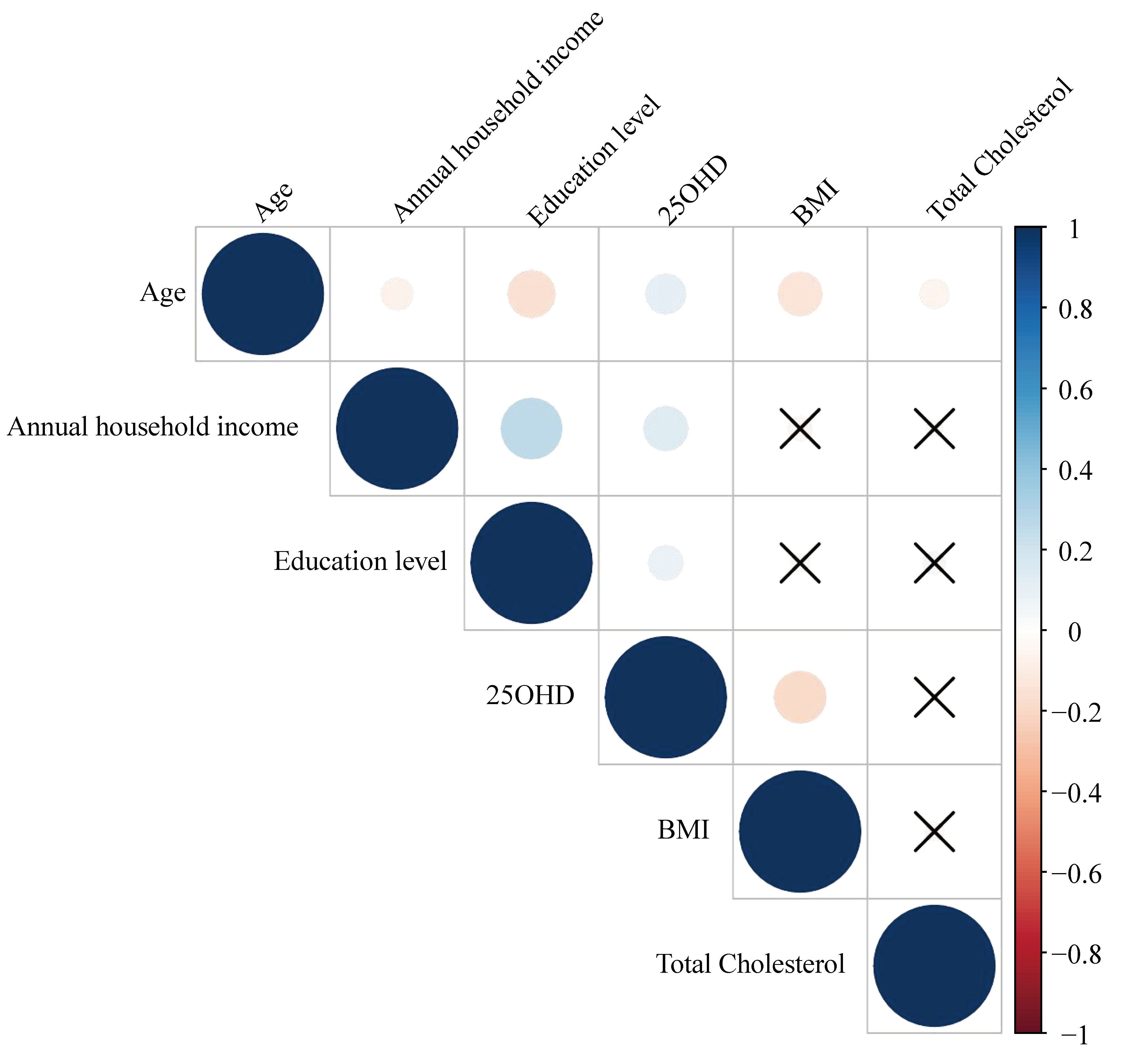


**Supplementary Figure 2.** Correlation analysis results of 25OHD and covariates.

**
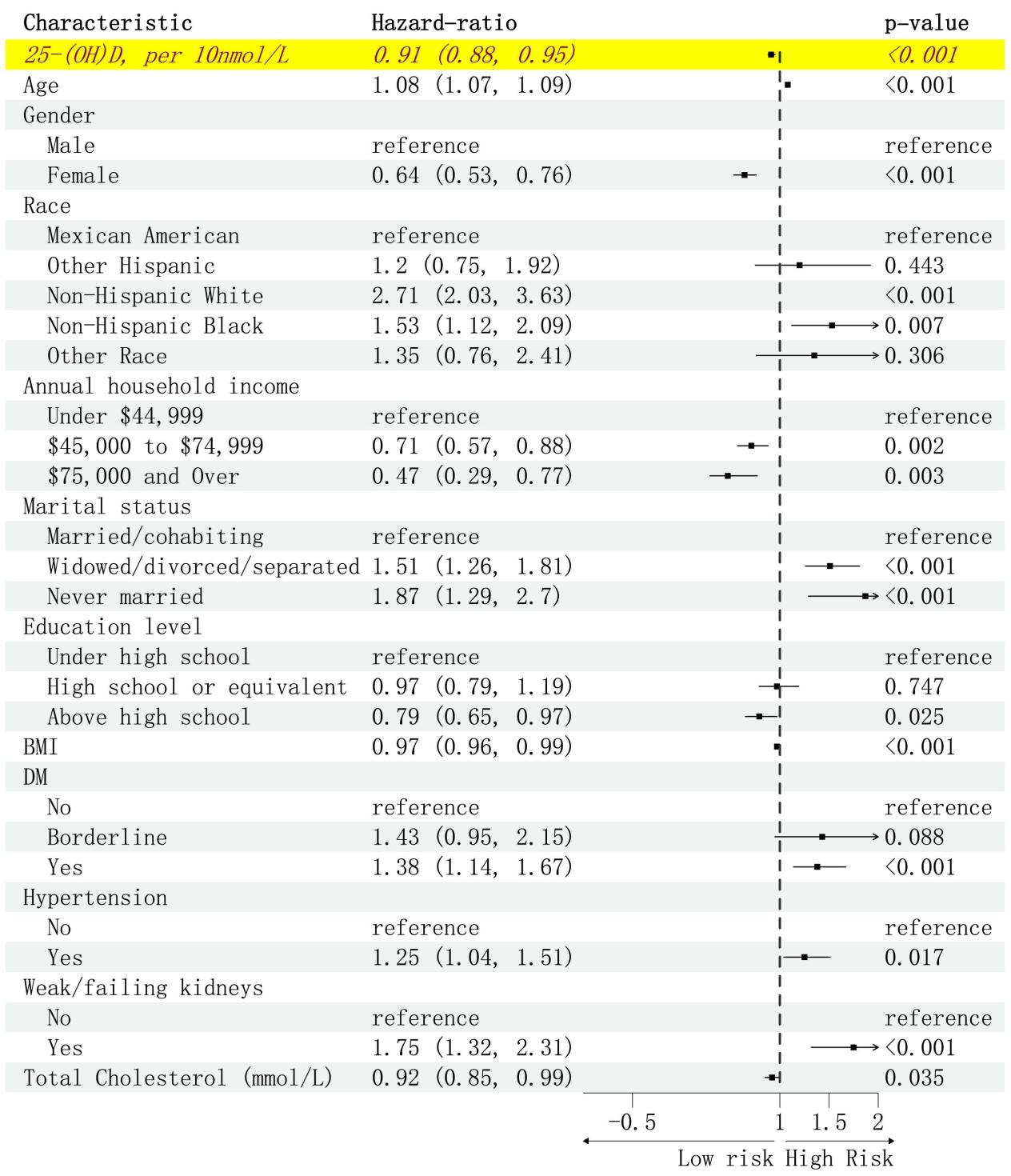
Supplementary Figure 3.** Hazard ratios (95% CI) of 25(OH)D and all covariates, unweighted. The model was adjusted for for age, sex, race, annual household income, marital status, education level, BMI, diabetes, hypertension, weak/failing kidneys, and total cholesterol.

**Supplementary Table 1.** Demographic characteristics of participants according to status, unweighted.

| **Characteristic** | **Assumed alive** | **Assumed deceased** | **p-value** |
| --- | --- | --- | --- |
|  | **(N=1704)** | **(N=586)** |  |
| Sex = female (%) | 1036 (60.8) | 301 (51.4) | **<0.001** |
| Age | 60.00 (49.00, 67.00) | 72.50 (63.00, 80.00) | **<0.001** |
| Race (%) |  |  | **<0.001** |
| Mexican American | 286 (16.8) | 60 (10.2) |  |
| Other Hispanic | 170 (10.0) | 25 ( 4.3) |  |
| Non-Hispanic White | 622 (36.5) | 333 (56.8) |  |
| Non-Hispanic Black | 511 (30.0) | 153 (26.1) |  |
| Other Race | 115 ( 6.7) | 15 ( 2.6) |  |
| 25OHD (nmol/L) | 61.58 (44.48, 80.95) | 55.70 (37.50, 72.90) | **<0.001** |
| Months of follow-up | 100.00 (49.00, 151.25) | 70.00 (34.00, 116.00) | **<0.001** |
| Annual household income (%) |  |  | **<0.001** |
| Under $44,999 | 1086 (63.7) | 460 (78.5) |  |
| $45,000 to $74,999 | 383 (22.5) | 109 (18.6) |  |
| $75,000 and Over | 235 (13.8) | 17 ( 2.9) |  |
| Marital status (%) |  |  | **<0.001** |
| Married/cohabiting | 975 (57.2) | 260 (44.4) |  |
| Widowed/divorced/separated | 577 (33.9) | 292 (49.8) |  |
| Never married | 152 ( 8.9) | 34 ( 5.8) |  |
| Education level (%) |  |  | **<0.001** |
| Under high school | 541 (31.7) | 264 (45.1) |  |
| High school or equivalent | 396 (23.2) | 152 (25.9) |  |
| Above high school | 767 (45.0) | 170 (29.0) |  |
| BMI | 30.10 (25.90, 35.23) | 28.49 (24.40, 32.78) | **<0.001** |
| Diabetes (%) |  |  | **0.005** |
| No | 1250 (73.4) | 389 (66.4) |  |
| Borderline | 55 ( 3.2) | 25 ( 4.3) |  |
| Yes | 399 (23.4) | 172 (29.4) |  |
| Hypertension = Yes (%) | 963 (56.5) | 410 (70.0) | **<0.001** |
| Weak/failing kidneys = Yes (%) | 120 ( 7.0) | 59 (10.1) | **0.024** |
| Total Cholesterol (mmol/L) | 4.97 (4.28, 5.69) | 4.89 (4.14, 5.74) | 0.089 |
| 25(OH)D = 25-hydroxyvitamin D; BMI = Body mass index. The bold values means statistical significance. | | | |

**Supplementary Table 2.** Demographic characteristics of participants according to 25(OH)D status, unweighted.

| **Characteristic** | **Deficiency** | **Insufficiency** | **Sufficiency** | **p-value** | |
| --- | --- | --- | --- | --- | --- |
|  | **(N=811)** | **(N=802)** | **(N=677)** |  | |
| Sex = female (%) | 501 (61.8) | 441 (55.0) | 395 (58.3) | **0.022** | |
| Age | 61.00 (51.00, 70.00) | 61.00 (51.00, 70.00) | 64.00 (54.00, 74.00) | **<0.001** | |
| Race (%) |  |  |  | **<0.001** | |
| Mexican American | 132 (16.3) | 141 (17.6) | 73 (10.8) |  | |
| Other Hispanic | 67 (8.3) | 84 (10.5) | 44 (6.5) |  | |
| Non-Hispanic White | 205 (25.3) | 370 (46.1) | 380 (56.1) |  | |
| Non-Hispanic Black | 376 (46.4) | 172 (21.4) | 116 (17.1) |  | |
| Other Race | 31 (3.8) | 35 (4.4) | 64 (9.5) |  | |
| 25OHD (nmol/L) | 37.30 (28.10, 43.40) | 61.65 (56.30, 67.85) | 90.95 (81.85, 106.45) | **<0.001** | |
| Months of follow-up | 102.00 (50.00, 157.50) | 100.00 (50.00, 147.00) | 73.00 (34.00, 121.00) | **<0.001** | |
| Annual household income (%) |  |  |  | **<0.001** | |
| Under $44,999 | 580 (71.5) | 554 (69.1) | 412 (60.9) |  | |
| $45,000 to $74,999 | 175 (21.6) | 164 (20.4) | 153 (22.6) |  | |
| $75,000 and Over | 56 (6.9) | 84 (10.5) | 112 (16.5) |  | |
| Marital status (%) |  |  |  | **0.004** | |
| Married/cohabiting | 399 (49.2) | 451 (56.2) | 385 (56.9) |  | |
| Widowed/divorced/separated | 333 (41.1) | 284 (35.4) | 252 (37.2) |  | |
| Never married | 79 (9.7) | 67 (8.4) | 40 (5.9) |  | |
| Education level (%) |  |  |  | **0.001** | |
| Under high school | 328 (40.4) | 267 (33.3) | 210 (31.0) |  | |
| High school or equivalent | 165 (20.3) | 209 (26.1) | 174 (25.7) |  | |
| Above high school | 318 (39.2) | 326 (40.6) | 293 (43.3) |  | |
| BMI | 31.54 (26.37, 36.74) | 29.40 (25.70, 34.06) | 28.35 (24.36, 32.70) | **<0.001** | |
| Diabetes (%) |  |  |  | **0.005** | |
| No | 550 (67.8) | 599 (74.7) | 490 (72.4) |  | |
| Borderline | 27 (3.3) | 34 (4.2) | 19 (2.8) |  | |
| Yes | 234 (28.9) | 169 (21.1) | 168 (24.8) |  | |
| Hypertension = Yes (%) | 507 (62.5) | 463 (57.7) | 403 (59.5) | 0.141 | |
| Weak/failing kidneys = Yes (%) | 62 (7.6) | 52 (6.5) | 65 (9.6) | 0.082 | |
| Total Cholesterol (mmol/L) | 4.91 (4.22, 5.66) | 4.99 (4.32, 5.72) | 4.91 (4.24, 5.72) | 0.299 | |
| 25(OH)D = 25-hydroxyvitamin D; BMI = Body mass index. The bold values means statistical significance. | | | | |  |

**Supplementary Table 3.** Demographic characteristics of participants according to 25(OH)D status, weighted.

| **Characteristic** | **Deficiency** | **Insufficiency** | **Sufficiency** | **p-value** | |
| --- | --- | --- | --- | --- | --- |
|  | **(N=2239422)** | **(N=3022926)** | **(N=2918140)** |  | |
| Sex = female (%) | 1442090 (64.4) | 1646153.9 (54.5) | 1715968.1 (58.8) | **0.013** | |
| Age (years) | 57.00 (47.00, 67.00) | 57.00 (46.00, 67.89) | 59.00 (50.00, 71.00) | **0.011** | |
| Race (%) |  |  |  | **<0.001** | |
| Mexican American | 195262 (8.7) | 234148.1 (7.7) | 119055.6 (4.1) |  | |
| Other Hispanic | 128342 (5.7) | 192435 (6.4) | 92935 (3.2) |  | |
| Non-Hispanic White | 1018946 (45.5) | 2096893 (69.4) | 2319296 (79.5) |  | |
| Non-Hispanic Black | 782276 (34.9) | 336725 (11.1) | 215557 (7.4) |  | |
| Other Race | 114594 (5.1) | 162724 (5.4) | 171295 (5.9) |  | |
| 25OHD (nmol/L) | 37.70 (28.80, 43.62) | 62.08 (56.45, 68.20) | 90.22 (81.80, 106.45) | **<0.001** | |
| Months of follow-up | 103.00 (53.00, 165.00) | 102.17 (49.17, 158.42) | 82.00 (39.00, 127.00) | **0.001** | |
| Annual household income (%) |  |  |  | **<0.001** | |
| Under $44,999 | 1511186 (67.5) | 1783927 (59.0) | 1489350 (51.0) |  | |
| $45,000 to $74,999 | 558394 (24.9) | 841601 (27.8) | 752875 (25.8) |  | |
| $75,000 and Over | 169841 (7.6) | 397398 (13.1) | 675914 (23.2) |  | |
| Marital status (%) |  |  |  | **0.025** | |
| Married/cohabiting | 1221820 (54.6) | 1797937 (59.5) | 1892518 (64.9) |  | |
| Widowed/divorced/separated | 813315 (36.3) | 1001340 (33.1) | 848351 (29.1) |  | |
| Never married | 204286 (9.1) | 223648 (7.4) | 177269 (6.1) |  | |
| Education level (%) |  |  |  | **0.018** | |
| Under high school | 704177 (31.4) | 675630 (22.4) | 657098 (22.5) |  | |
| High school or equivalent | 482939 (21.6) | 898744 (29.7) | 867494 (29.7) |  | |
| Above high school | 1052305 (47.0) | 1448552 (47.9) | 1393547 (47.8) |  | |
| BMI | 31.89 (26.37, 37.10) | 29.12 (25.19, 33.69) | 28.30 (24.10, 32.30) | **<0.001** | |
| Diabetes (%) |  |  |  | **0.034** | |
| No | 1615572 (72.1) | 2389387 (79.0) | 2337185 (80.1) |  | |
| Borderline | 76124 (3.4) | 107290 ( 3.5) | 69473 (2.4) |  | |
| Yes | 547725 (24.5) | 526249 (17.4) | 511481 (17.5) |  | |
| Hypertension = Yes (%) | 1309926 (58.5) | 1543128 (51.0) | 1548712 (53.1) | 0.141 | |
| Weak/failing kidneys = Yes (%) | 157792 (7.0) | 175721 (5.8) | 223938 (7.7) | 0.514 | |
| Total Cholesterol (mmol/L) | 4.97 (4.27, 5.69) | 4.99 (4.34, 5.72) | 5.09 (4.39, 5.84) | 0.264 | |
| 25(OH)D = 25-hydroxyvitamin D; BMI = Body mass index. The bold values means statistical significance. | | | | |  |

**Supplementary Table 4.** Hazard ratio (95% CI) of all cause mortality according to 25(OH)D status.

| **Characteristic** | **Unweighted** | | **Weighted** | |
| --- | --- | --- | --- | --- |
|  | **HR (95% CI)** | **p-value** | **HR (95% CI)** | **p-value** |
| Deficiency (25OHD <50 nmol/L) | reference |  | reference |  |
| Insufficiency (50 nmol/L≤25OHD ≤75 nmol/L) | 0.65 (0.53, 0.80) | **<0.001** | 0.64 (0.50, 0.83) | **<0.001** |
| Sufficiency (25OHD >75 nmol/L) | 0.65 (0.52, 0.81) | **<0.001** | 0.60 (0.44, 0.80) | **<0.001** |
| HR = Hazard ratio; CI = Confidence interval; 25(OH)D = 25-hydroxyvitamin D. The bold values means statistical significance. HRs were adjusted for age, sex, race, annual household income, marital status, education level, BMI, diabetes, hypertension, weak/failing kidneys, and total cholesterol. | | | | |
